# Atom-to-atom interactions for atomic layer deposition of trimethylaluminum on Ga-rich GaAs(001)-4 × 6 and As-rich GaAs(001)-2 × 4 surfaces: a synchrotron radiation photoemission study

**DOI:** 10.1186/1556-276X-8-169

**Published:** 2013-04-12

**Authors:** Tun-Wen Pi, Hsiao-Yu Lin, Ya-Ting Liu, Tsung-Da Lin, Gunther K Wertheim, Jueinai Kwo, Minghwei Hong

**Affiliations:** 1 National Synchrotron Radiation Research Center, Hsinchu 30076, Taiwan; 2 Department of Physics, National Tsing Hua University, Hsinchu 30013, Taiwan; 3 Graduate Institute of Applied Physics and Department of Physics, National Taiwan University, Taipei 10617, Taiwan; 4 Woodland Consulting, 175 Woodland Ave, Morristown, NJ, 07960, USA

**Keywords:** GaAs, Al_2_O_3_, Atomic layer deposition, Synchrotron radiation photoemission, 73.90. + f, 79.60.Jv

## Abstract

High-resolution synchrotron radiation photoemission was employed to study the effects of atomic-layer-deposited trimethylaluminum (TMA) and water on Ga-rich GaAs(001)-4 × 6 and As-rich GaAs(001)-2 × 4 surfaces. No high charge states were found in either As 3*d* or Ga 3*d* core-level spectra before and after the deposition of the precursors. TMA adsorption does not disrupt the GaAs surface structure. For the (4 × 6) surface, the TMA precursor existed in both chemisorbed and physisorbed forms. In the former, TMA has lost a methyl group and is bonded to the As of the As-Ga dimer. Upon water purge, the dimethylaluminum-As group was etched off, allowing the now exposed Ga to bond with oxygen. Water also changed the physisorbed TMA into the As-O-Al(CH_3_)_2_ configuration. This configuration was also found in 1 cycle of TMA and water exposure of the (2 × 4) surface, but with a greater strength, accounting for the high interface defect density in the mid-gap region.

## Background

Atomic layer deposition (ALD) facilitates the deposition of a dielectric oxide onto a GaAs surface. The process differs from the one used for the deposition of ALD oxide on Si, where an OH group on the semiconductor is required to initiate the deposition. Bonding of the oxide on the III-V semiconductor is accessible to investigation with high-resolution synchrotron radiation photoemission. It provides unprecedented, precise information about the interfacial electronic structure. This information is vital because the interfacial trap density (*D*_it_) governs the performance of GaAs-based devices. In order to obtain consistent information, the III-V surface must be free of impurities, such as oxygen, and other defects prior to the ALD process. Only when this condition is satisfied will the true interfacial electronic structure be revealed.

The attempt to prepare a clean GaAs(001) surface has generally been patterned on the procedure used to obtain a clean Si(001) surface. That neglects the fundamental difference between the surface properties and reconstruction of a III-V semiconductor and an elemental one like Si. The reconstructed Si(001)-2 × 1 surface consists of rows of buckled dimers, with charge transfer between the tilted atoms, and is rich in dangling bonds that trap impurities. Surface pretreatment is required prior to a final anneal in an ultra-high-vacuum end station prior to synchrotron radiation photoemission (SRPES) measurements. The pretreatment due to Ishizaka and Shiraki [1] has come into general use. It leaves a thin oxide film on a clean Si surface that is readily removed by annealing in vacuum [2,3]. The effectiveness of this procedure has been demonstrated in [2], which shows the analysis of 2*p* core-level data from a clean reconstructed Si(001) surface. The photoemission spectra from the first three surface layers labeled S(0), S(1), and S(2) are identified and have intensities consistent with the expected escape depth.

For multi-element (In)GaAs, a common method of surface pretreatment prior to in-vacuum annealing is As capping [4] by thermal annealing in As_2_ flux [5], followed by a chemical rinse [6]. Subsequent in-vacuum annealing of these samples removes the more volatile As and produces an oxygen-free surface, but one that does not have the desired surface Ga/As ratio. It turns out to be low, say 0.73 [4], compared with an untreated sample, say 1.26 (not shown). The process has clearly changed the surface stoichiometry as well as the surface reconstruction. To be specific, ALD of Al_2_O_3_ with trimethylaluminum (TMA) and water on the treated GaAs(001) with ammonia or ozone often left As-As dimers at the interface, resulting in significant frequency dispersion in the *C*-*V* characteristic curve [7-9]. This conventional cleaning process does not reproduce the clean reconstructed surface and must be adjudged a failure. The resulting uncertainty regarding the chemistry and reconstruction of the surface prevents an understanding of the nature of the interaction with adsorbates and stands in the way of systematic improvements. It impacts both work on the interfacial electronic structure of high-*κ* dielectric oxides/(In)GaAs [10-12] and spintronics based on Fe_3_Si/GaAs [13,14].

In this work, we present a high-resolution core-level SRPES investigation of the electronic structure of the clean, Ga-rich GaAs(001)-4 × 6 surface, followed by the characterization of the surface after 1 cycle of ALD of, first, TMA and then water H_2_O onto the TMA-covered surface. For comparison, we also present the data of 1 cycle of TMA and H_2_O on As-rich GaAs(001)-2 × 4. We note that the ALD precursors were exposed onto a surface with a long-range order, a condition of that has not been previously achieved in work with GaAs.

## Method

The samples were fabricated in a multi-chamber growth/analysis system, which includes a GaAs-based molecular beam epitaxy (MBE) chamber, an ALD reactor, and many other functional chambers [15,16]. These chambers are connected via transfer modules, which maintain ultra-high vacuum of 10^−10^ Torr. Thus, pristine surfaces were obtained during the sample transfer. MBE was employed to grow Si-doped GaAs (1 to 5 × 10^17^ cm^−3^) onto 2-in. n-GaAs(100) wafers. ALD was employed to high *κ* dielectrics on freshly MBE-grown GaAs. The samples were transferred *in vacuo* into a portable module kept at 2 × 10^−10^ Torr and transported to the National Synchrotron Radiation Research Center located in Taiwan for SRPES measurements. Photoelectrons were collected with a 150-mm hemispherical analyzer (SPECS, Berlin, Germany) in an ultra-high vacuum chamber with a base pressure of approximately 2 × 10^−10^ Torr. The overall instrumental resolution was better than 60 meV, and the binding energy was established in accordance with the Fermi edge of Ag.

## Results and discussion

**Figure 1:**
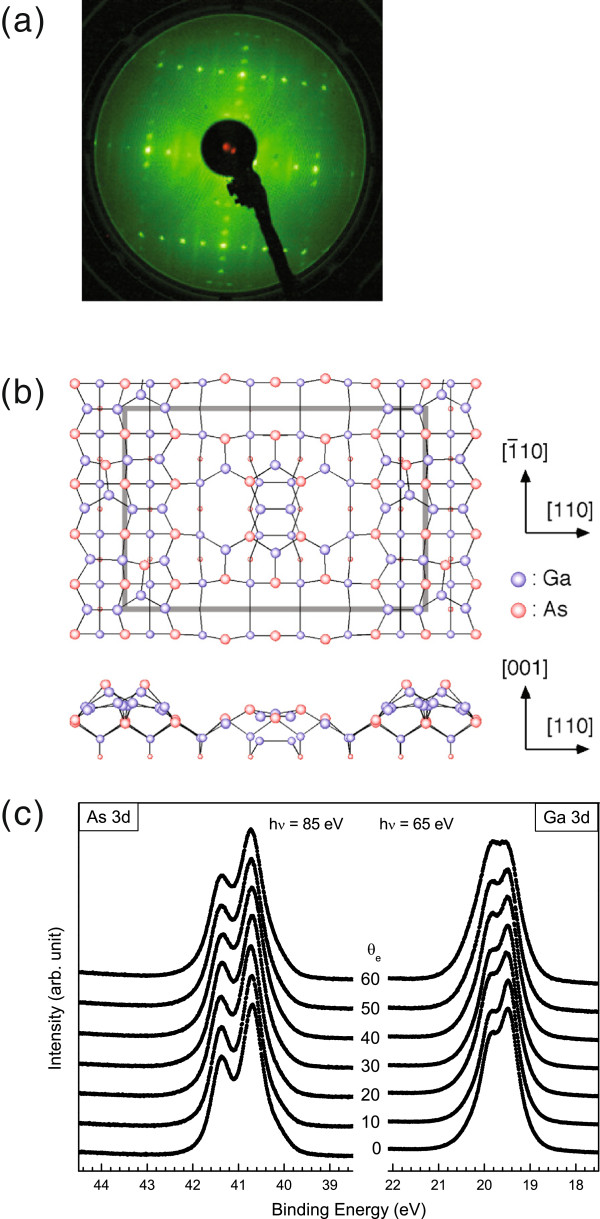
**Basics of the GaAs(001)-4 × 6 surface.** (**a**) A LEED pattern using an electron energy of 51 eV, (**b**) atomic structure proposed by Ohtake et al. (adapted from [17]. copyright 2004 American Physical Society), and (**c**) As 3*d* and Ga 3*d* core-level photoemission spectra with various emission angles (*θ*_e_).

The surface reconstruction of GaAs(001) was first checked with reflection high-energy electron diffraction in the molecular beam epitaxial growth chamber and then verified with low-energy electron diffraction (LEED) in the photoemission chamber. The LEED pattern is shown in Figure 1a. It consists of sharp 4 × 6 spots and third-order streaks along the [110] direction. The streaking pattern indicates that the surface contains small domains of (6 × 6) or c(8 × 2) reconstruction. The low background intensity indicates that the surface is smooth with a great long-range order. Recently, Ohtake et al. proposed that for the GaAs(001)-4 × 6 surface, the unit cell is composed of four As atoms in the faulted position, two tilted As-Ga dimers and two Ga-Ga dimers in the subsurface layer [17]. The atomic structure of the Ohtake model is shown in Figure 1b.

Figure 1c displays the As 3*d* and Ga 3*d* core-level spectra of a clean Ga-rich n-GaAs(001)-4 × 6 surface taken in various angles from the normal emission to 60° off-normal emission. The excitation photon energies were set at 85 and 65 eV for As and Ga states, respectively. The estimated escape depth is approximately 0.3 to 0.5 nm. A visual inspection of the As (Ga) 3*d* photoemission data identifies a feature bulged out at low (high) binding energy, suggesting that the line shape contains components in addition to the main bulk line. In fact, deconvolution of the As 3*d* core-level spectrum shows four components. Accordingly, we set up a model function with four spin-orbit pairs as well as a power-law background and a plasmon- or gap-excitation-energy loss tail. The background and loss tail are represented by least squares adjustable parameters that are included in the model function. The background is represented by four parameters: a constant, a slope, and a power-law that is quite successful in representing the degraded electrons from shallower levels. In the energy range of the 3*d* spectra, the loss tail is almost entirely due to electron-hole pair excitations in the semiconductor. In GaAs, there are none that are smaller than the 1.42-eV bandgap, which implies that almost all of the line structure remains unaffected by the loss tail. Background subtraction prior to fitting meets with a fundamental objection. It destroys the statistical relationship between the number of counts in the data point and its uncertainty, preventing *χ*^2^ from reaching unity for a perfect fit and interfering with the assessment of the quality of the fit.

The fact that the resolved components in the deconvolute exhibit nearly equal widths suggests that the lifetime is the same for all components. The residual differences in width are presumably due mainly to small differences in the phonon or inhomogeneous broadening of bulk and surface components. It is worth noting that a reliable least squares adjustment is readily obtained provided the model function has a multi-parameter global minimum. A multitude of unconstrained width parameters tend to produce local minima defining erroneous, unphysical parameter values. The width parameters were accordingly constrained as needed.

**Figure 2:**
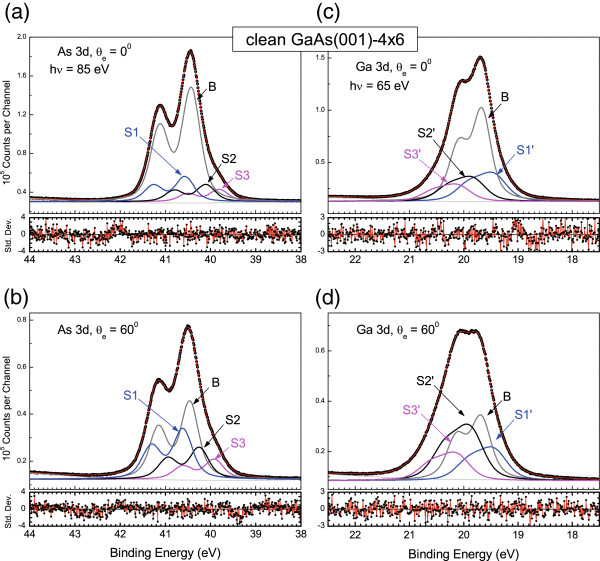
**Analysis of the core-level spectra for the clean Ga-rich GaAs(001)-4 × 6 surface.** (**a**) As 3*d* state, *θ*_e_ = 0°, (**b**) As 3*d* state, *θ*_e_ = 60°, (**c**) Ga 3*d* state, *θ*_e_ = 0°, and (**d**) Ga 3*d* state, *θ*_e_ = 60°.

The representative fit to the As and Ga 3*d* states of the clean GaAs(001)-4 × 6 surface are shown in Figure 2. The numerical results were obtained using the nonlinear Marquardt algorithm. The surface core-level shifts (SCLSs) of the Ga 3*d* state for the S1', S2', and S3' components relative to the bulk at 19.58 eV are −0.302, +0.251, and +0.613 eV, respectively. The Gaussian widths of the bulk and surface are 0.33 and 0.45 eV, respectively. For the As 3*d* state, the S1, S2, and S3 components relative to the bulk located at 40.43 eV (the 3*d*_5/2_ state) were found to be +0.159, −0.249, and −0.599 eV, respectively. A ‘+’ or ‘−‘ sign indicates a shift towards a higher or lower binding energy, respectively. The Gaussian width is about 0.31 eV. The lifetime is 0.22 eV. In Figure 2b,d, the change in intensity of the components at 60° emission angle is displayed, clearly identifying the surface components. The smallest As component, S3, is most likely associated with the As in the tilted As-Ga dimers in the defaulted terrace. The shifted magnitude of component S3 is the greatest among those reported in the literature, suggesting that the tilted angle of the dimer is great so as to cause a large charge transfer.

**Figure 3:**
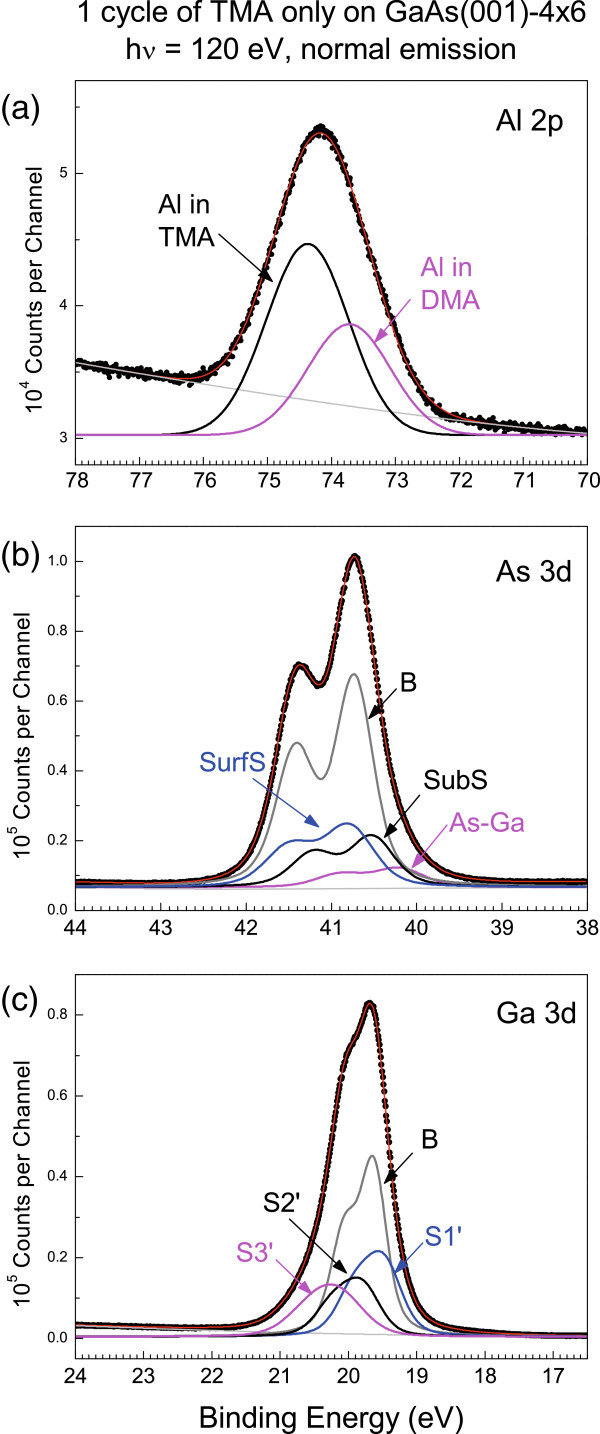
**Analysis of the core-level spectra influenced by 1 cycle of TMA-only exposure.** (**a**) Al 2*p*, (**b**) As 3*d*, and (**c**) Ga 3*d* states.

Figure 3 displays a fit to the TMA-exposed surface prior to exposure to H_2_O. As shown in Figure 3a, two Al 2*p* states are well resolved with an energy separation of 0.650 eV. The one with lower binding energy is associated with a charge transfer from As to Al. This is possible when a methyl ligand is replaced by a direct bond to an As atom. Considering that the GaAs(001)-4 × 6 surface is ‘As-terminated’ and component S3 shows a negative SCLS, we assumed that dimethylaluminum (DMA) bonds with the dangling bond of the As in the As-Ga dimer.

Because the high-binding-energy Al 2*p* state remains in the same position and with similar line width after the subsequent water purge, the TMA precursor must have maintained the Al in the molecular charge state while residing on the surface. That indicates that this TMA does not form a bond with a surface atom. That is in agreement with the absence of a new surface As level and leads to the conclusion that the TMA is *physisorbed* on the S1 As atoms.

For the As 3*d* core-level spectrum, the TMA-exposed surface reveals only minor changes from the clean surface. First, the widths of both top-surface S1 and S3 components are 15% to 20% broader than the subsurface S2 component. Second, the SCLS of the S1 component becomes 0.056 eV without changing the strength. Third, the intensity of the S3 component slightly decreases concurrently with a slight increase of the S2 intensity. Because the Al in DMA bonds with S3 As atom, this As underneath the Al behaves as a subsurface atom. Consequently, the energy position coincides with that of the S2 state, thereby increasing the S2 intensity slightly.

Figure 3c presents a fit to the Ga 3*d* core-level spectrum. The Ga 3*d* states remain virtually unaltered, indicating that the TMA precursor has not disturbed the Ga layer.

**Figure 4:**
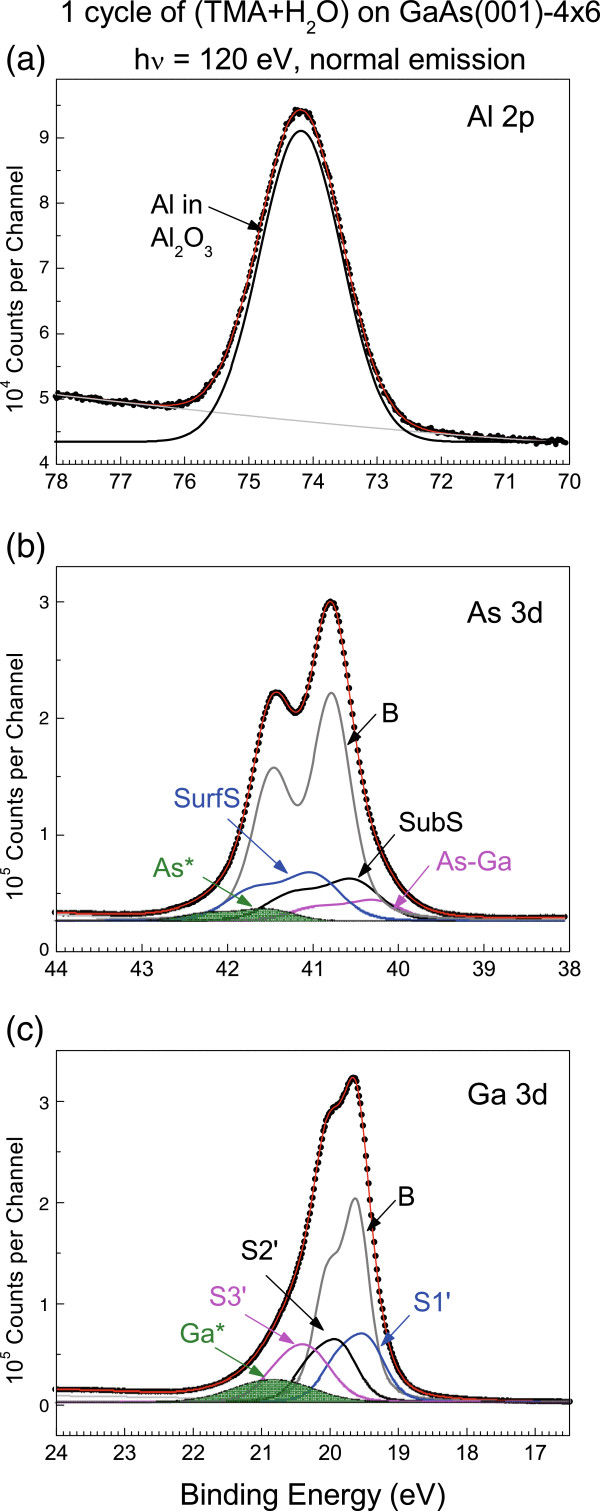
**Analysis of the core-level spectra influenced by 1 cycle of TMA and H**_**2**_**O exposure.** (**a**) Al 2*p*, (**b**) As 3*d*, and (**c**) Ga 3*d* states.

Figure 4 displays a fit to the spectra after 1 cycle of TMA and H_2_O purges. The Al 2*p* state now exists as a single peak without any sign of the component identified with DMA. This suggests that the H_2_O precursor has etched off the attached Al-(CH_3_)_2_ species that bonded to the As in the As-Ga dimer. Removal of the As atoms exposes the previously dimerized Ga atom which now becomes oxidized as shown in Figure 4c, where the oxidized Ga* state appears with SCLS of +0.892 eV. Note that the area of the S2 state retains the magnitude in the clean surface.

Figure 4b exhibits As-induced states labeled as As* with SCLSs of +0.680 eV. The energy separation of the As* and S1 states is 0.432 eV, which remains constant in the greater cycles of deposition (not shown), indicating that the As* state originated from the S1 As atoms. Because the SCLS of the As* state becomes more positive than that of the S1 state, under the influence of water, the adsorbed TMA precursor must undergo a change of bonding configuration to become a charge acceptor for the affiliated As atom. Because no similar Al-X state appears in the Al 2*p* core-level spectrum, water then affects the TMA molecule that is physisorbed on As in a way that allows the interfacial S1 As to become an As-O-Al configuration, where the surface is further terminated with a hydroxyl group.

**Figure 5:**
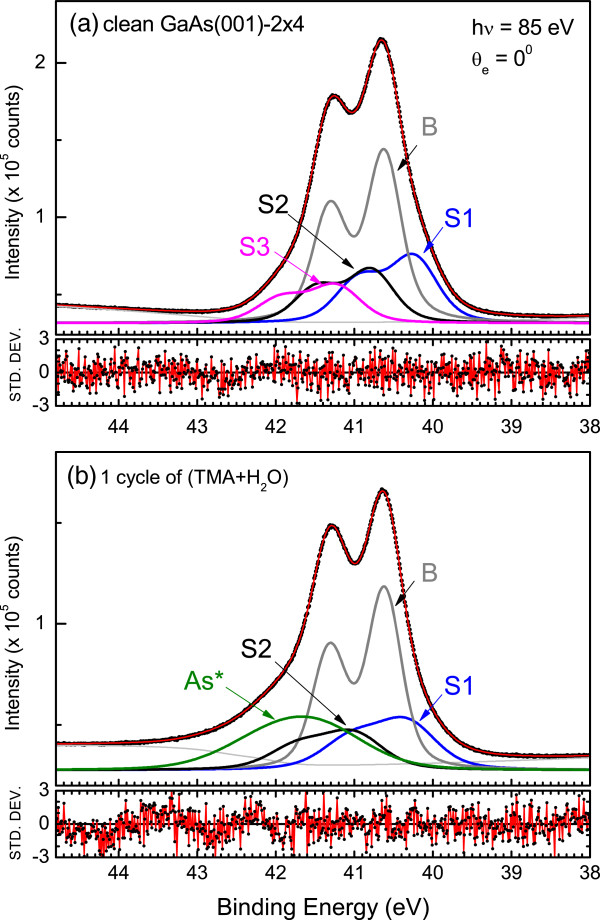
**Analysis of the As 3*d *core-level spectra of As-rich GaAs(001)-2 × 4.** (**a**) The clean surface and (**b**) the surface after exposure to 1 cycle of TMA and H_2_O purges.

Figure 5a shows a fit to the As 3*d* core-level spectrum for the clean As-rich GaAs(001)-2 × 4 surface. The β2(2 × 4) model is commonly believed to represent the surface reconstruction, where the top surface layer is characterized as two rows of As-As dimers separated by itself from an As-As dimer located in the third layer. As can be seen in Figure 5a, three surface components were resolved. With reference to an off-normal spectrum (not shown), both the S1 and S3 components are identified with the surface As-As dimers because of the intensity enhancement. In fact, components S3 and S1 are associated with the As-As dimers in the first and third layers, respectively. Figure 5b displays a fit to this surface covered with 1 cycle of (TMA + H_2_O) purges. The S3 component has been replaced with an induced As* component with a shift from the bulk of +0.707 eV. Clearly, the outmost surface As dimer bonds are passivated. The intensity of the As* component in the As-rich surface is greater than that in the Ga-rich surface. The greater intensity of the As* state in the GaAs(001) 2 × 4 surface results in a greater value of *D*_it _in the mid-gap and inferior device performances, as shown in [18] and [19], respectively.

## Conclusions

We have presented a microscopic view of *in situ* atomic layer deposition of TMA and H_2_O precursors on atomically clean GaAs(001) surfaces in both 4 × 6 and 2 × 4 reconstructions. For the Ga-rich 4 × 6 surface, the precursors partially and selectively bond with the surface atoms without disturbing the atoms in the subsurface layer. TMA is dissociative on As in the As-Ga dimer but is physisorbed on As that is threefold Ga-coordinated. Water drastically alters the TMA-covered surface, etching off the DMA along with its As, resulting in Ga-O bonding for the subsequent deposition of Al_2_O_3_. At the same time, it transforms the configuration of the physisorbed TMA to bond strongly with As. On the As-rich 2 × 4 surface, 1 cycle of TMA and H_2_O entirely passivates the surface As dimer bonds.

## Abbreviations

ALD: Atomic layer deposition; Dit: Interfacial trap density; DMA: Dimethylaluminum; LEED: Low-energy electron diffraction; SCLS: Surface core-level shift; SRPES: Synchrotron radiation photoemission; TMA: Trimethylaluminum.

## Competing interests

The authors declare that they have no competing interests.

## Authors’ contributions

All authors have contributed to the final manuscript of the present work. MH, JK, and TWP defined the research topic. TDL provided the GaAs sample. YTL prepared the precursor-purged interfaces. HYL acquired the photoemission data. TWP wrote the paper. GKW and MH provided critical comments on the draft manuscript. All authors read and approved the final manuscript.

## Authors’ information

TWP is a research scientist at the National Synchrotron Radiation Research Center, Hsinchu, Taiwan. GKW is the President of Woodland Consulting in the USA. JK is a full professor in the Department of Physics, National Tsinghua University, Hsinchu, Taiwan. MH is a full professor in the Graduate Institute of Applied Physics and Department of Physics, National Taiwan University, Taipei, Taiwan. TDL is a postdoctoral fellow in the Graduate Institute of Applied Physics and Department of Physics, National Taiwan University, Taipei, Taiwan. HYL is a graduate student in the Department of Physics, National Tsinghua University, Hsinchu, Taiwan. YTL is a graduate student in the Department of Materials Science, National Tsinghua University, Hsinchu, Taiwan.
